# The clinical significance of TAT, PIC, TM, and t-PAIC in vascular events of BCR/ABL-negative myeloproliferative neoplasms

**DOI:** 10.1007/s10238-024-01371-7

**Published:** 2024-05-22

**Authors:** Kangle Huang, Qiuyu Mo, Chushu Liao, Shan Feng, Guanghua Liu, Duanfeng Jiang, Ping Lei

**Affiliations:** 1 Department of Hematology, The Hunan Provincial People’s Hospital, The First Affiliated Hospital of Hunan Normal University, Jiefang West Road, No. 61, Changsha, 410005 China; 2 Department of Oncology, The Fourth Affiliated Hospital of Guangxi Medical University, Liuzhou, 545007 Guangxi China; 3 Department of Hematology, The Fourth Affiliated Hospital of Guangxi Medical University, Heping Road, No. 156, Liuzhou, 545007 Guangxi China; 4 Department of Hematology, The Second Affiliated Hospital of Hainan Medical University, Haikou, 570311 China

**Keywords:** Myeloproliferative neoplasms, Hemorrhagic events, Thrombotic events, Thrombin-antithrombin complex (TAT), Plasmin-α2-plasmininhibitor complex (PIC), Thrombomodulin (TM), Tissue plasminogen activator-inhibitor complex (t-PAIC)

## Abstract

Predicting the likelihood vascular events in patients with BCR/ABL1-negative myeloproliferative neoplasms (MPN) is essential for the treatment of the disease. However, effective assessment methods are lacking. Thrombin-antithrombin complex (TAT), plasmin-α_2_- plasmininhibitor complex (PIC), thrombomodulin (TM), and tissue plasminogen activator-inhibitor complex (t-PAIC) are the new direct indicators for coagulation and fibrinolysis. The aim of this study was to investigate the changes of these four new indicators in thrombotic and hemorrhagic events in BCR/ABL1-negative MPN. The study cohort of 74 patients with BCR/ABL negative myeloproliferative disorders included essential thrombocythemia, polycythemia vera, and primary myelofibrosis (PMF). A panel of 4 biomarkers, including TAT, PIC, TM, and t-PAIC were determined using Sysmex HISCL5000 automated analyzers, whereas fibrin/fibrinogen degradation products (FDP), D-dimer and Antithrombin III (ATIII) were analyzed using Sysmex CS5100 coagulation analyzer. A total of 24 (32.4%) patients experienced thrombotic events and hemorrhagic events occurred in 8 patients (10.8%). Compared to patients without hemorrhagic-thrombotic events, patients with thrombotic events had higher fibrinogen (FIB) level, FDP level and lower ATIII activity, while patients with hemorrhagic events had lower white blood cell count and hemoglobin level, higher FDP level (*P* < 0.05). Patients with a JAK2V617F mutation were more likely to experience thrombotic events (*P* < 0.05). In addtion, patients with thrombotic events had higher TAT, PIC, TM, and t-PAIC levels than patients without hemorrhagic-thrombotic events (*P* < 0.05), whereas patients with hemorrhagic events had a lower median value in TAT and TM (no statistical difference, *P* > 0.05). Patients with higher TAT, TM and t-PAIC were more likely to experience thrombotic events (*P* < 0.05), and only TAT was positively correlated with thrombotic events (Spearman  *r* =0.287, *P* = 0.019). TAT, PIC, TM, and t-PAIC combined with ATIII and FDP have a certain value for predicting thrombosis in patients with BCR/ABL1-negative MPN. These 6 parameters are worth further exploration as predictive factors and prognostic markers for early thrombotic events.

## Introduction

Myeloproliferative neoplasms (MPN) are clonal disorders of the hematopoietic stem cell characterized by a significant increase in one or more lineages of myeloid cells [1]. Classical BCR/ABL-negative MPN are a heterogeneous group of hematologic malignancies, including essential thrombocythemia (ET), polycythemia vera (PV), and primary myelofibrosis (PMF) [2]. In these disorders, thrombotic, vascular, and bleeding complications are the most common causes of morbidity and mortality [3]. Approximately 45% of all deaths in PV patients are caused by cardiovascular events [4]. MPN are the most common etiologies of primary splanchnic vein thrombosis (SVT), including mesenteric, splenic, or portal vein thrombosis (PVT), with about 30–40% of SVT being caused by MPN [5]. All types of MPN have been reported to cause vein thrombosis, though the most frequent appears to be PV, followed by ET and PMF [6]. The primary goal of treatment, especially those with PV and ET, is to prevent thrombotic complications typically through antiplatelet therapy and/or cytoreduction [6].

Although several patient-, disease-, and genome-related factors that affect the risk of thrombosis have been identified, there is are no sufficiently accurate laboratory investigation to assess potential procoagulant states and predict the thrombotic risks [7]. Excessive proliferation of hematopoietic precursors in the bone marrow of MPN patients leads to the release of a large number of mature blood cells into the circulation [8]. Erythrocytosis can cause venous or arterial thrombosis by directly increasing whole-blood viscosity, and leukocyte counts of > 15 × 10^9^/L in PV and > 11 × 10^9^/L in ET have been independently associated with an increased risk of thrombosis [9]. The factors significantly related to arterial thrombosis were age, increased white blood cells (WBC), general cardiovascular risk factors, JAK2V617F, and high molecular risk mutations, while only history of previous thrombosis, particularly prior venous thrombosis, was predictive of venous events [10]. The risk of total thromboses was accurately predicted by the international prognostic score for thrombosis in essential thrombocythemia (IPSET) score. The IPSET-revised and/or IPSET is recommended by recent guidelines [11], including those from the European Leukemia Net and the National Comprehensive Cancer Network, as the most appropriate risk scoring system(s) in patients with ET [12, 13]. However, no score has been specifically developed to assess thrombotic risk in PMF, reflecting difficulties and variability in the management of this condition by hematologists [14]. Guglielmelli et, al validated that IPSET score can be conveniently used for thrombosis risk stratification in patients with pre-PMF [10]. These results suggested that IPSET score might be used for thrombosis prediction in patients with BCR-ABL-negative MPN.

IPSET-thrombosis scores are useful for identifying patients at high risk of arterial thrombosis, whereas they fail to predict venous thrombosis [15]. The revised IPSET-thrombosis identified four risk categories based on three adverse variables (thrombosis history, age > 60 years and JAK2V617F). However, both IPSET-thrombosis and revised IPSET-thrombosis scoring systems failed to predict venous thrombosis [10]. The revised IPSET-thrombosis needs confirmation in prospective studies, especially in terms of risk-adapted therapy [16]. Furthermore, it has not been addressed yet whether the available scores for ET may be informative in patients with PMF or PV as well; this question was one of the objective of current study. Thrombosis in MPN has been linked to hypercoagulable state, and utilization of abnormal coagulation and fibrinolysis as surrogate biomarkers of thrombosis has been suggested [17, 18]. Conventional coagulation testing only measures time to clot formation and cannot reliably correlate thrombotic and hemorrhagic events [7]. Measurement of coagulation and fibrinolysis processes such as thromboelastography, thrombin, and fibrin generation may provide a more thorough assessment of hemostatic function [7]. Thrombin-antithrombin complex (TAT), plasmin-α_2_- plasmininhibitor complex (PIC), thrombomodulin (TM), and tissue plasminogen activator-inhibitor complex (t-PAIC) are new indicators which directly reflect the fibrinolysis and the activity of the endothelial system [19]. The TAT is recognized as a marker of activation of the coagulation system, PIC is an indicator of activation of the fibrinolysis system, TM can monitor the function of endothelial cells, and t-PAIC is a fibrinolytic marker. These are important markers in the process of venous thrombosis, which can be significantly elevated before thrombus [20].

The whole blood coagulation, including Antithrombin III (ATIII), D-dimer and fibrin/fibrinogen degradation products (FDP), the four new indicators TAT, PIC, TM and t-PAIC are laboratory parameters which reflect the hemostatic process, including the clot formation, stabilization, and dissolution. In this study, we provide a new perspective to the current evidence of the role of the four new indicators in MPN, particularly the relationship with vascular events.

## Materials and methods

### Patient involvement

**Fig. 1:**
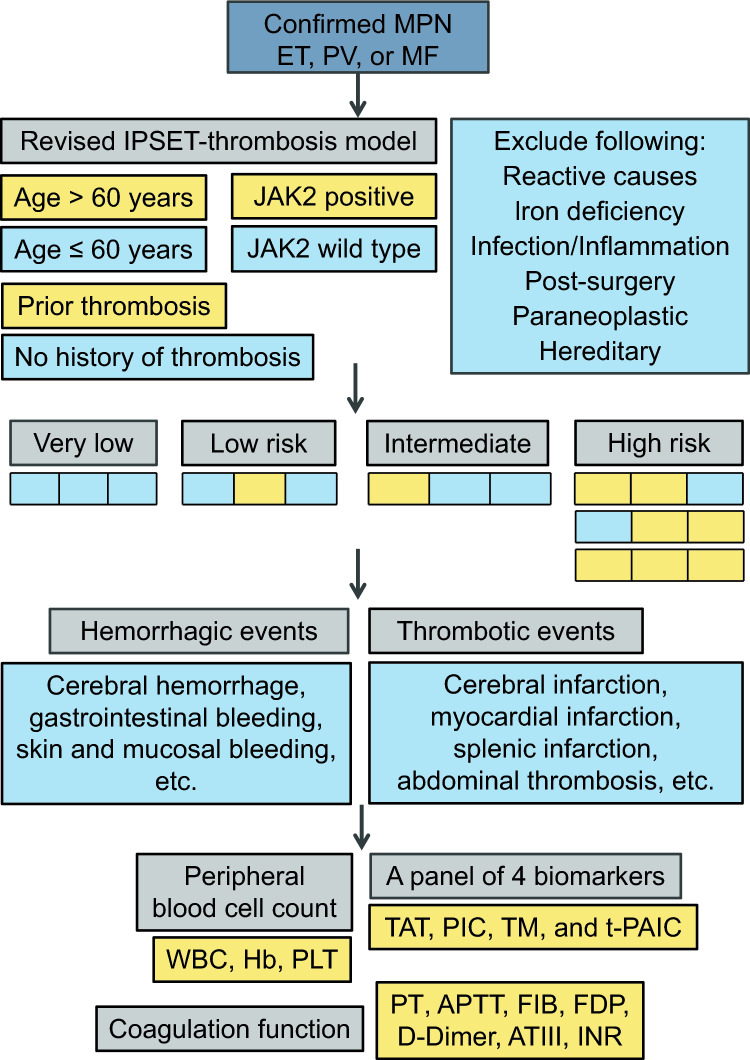
Our approach to MPN-associated vascular events

Clinical data of 74 patients, who were diagnosed with ET, PV, and PMF according to 2016 WHO criteria [11, 21] between September 2020 and July 2023, were retrospectively analyzed. Among them, patients with reactive causes, iron deficiency, infection, inflammation, post-surgery, paraneoplastic, hereditary, or underlying vascular diseases were excluded (Fig. 1). This study was approved by the Medical Ethics Committee of the First Affiliated Hospital of Hunan Normal University, and was conducted in accordance with the principles of the Declaration of Helsinki.

### Biomarker determination

After informed consent was obtained, venous blood samples were collected from all patients and centrifuged at 3000 g for 15 min to separate plasma. The TAT, PIC, TM, and t-PAIC test kits were provided by Sysmex Corporation. The measurements were based on chemiluminescence enzyme immunoassay method and performed on the Sysmex HISCL5000 automated analyzers. The ATIII, D-dimer and FDP were determined using Sysmex CS5100 coagulation analyzer.

### Statistical analysis

Continuous variables were were represented by medians and ranges (first and third quantiles), and categorical variables were presented as frequencies and percentages. The differences between the groups were compared using unpaired *t* test or Mann–Whitney *U* test for continuous variables, the *χ*^*2*^ test for categorical variables. Correlation between values was assessed by means of the Spearman rho correlation test. *P* values of less than 0.05 were considered statistically significant. Analyses were performed using IBM SPSS Statistics 19.0 and GraphPad Prism 8.0.

## Results

### Clinical features of patients with BCR/ABL-negative MPN

This study included a total of 74 BCR/ABL-negative MPN patients, including 28 ET (37.8%), 21 PV (28.4%), and 25 PMF (33.8%) patients. The patient clinical and laboratory characteristics at diagnosis are showed in Table 1. The median age of all patients at diagnosis was 65 years (range, 32–84). Among them, 40.5% were under the age of 60 years; and 40.5% were male. According to the International Working Group for myeloproliferative neoplasm (MPN) Research and Treatment (IWG-MRT) [22], the revised risk stratification scheme (revised IPSET-thrombosis model, R-IPSET) include four categories: ‘very low risk' (no thrombosis history, age < 60 years and JAK2-wide type); ‘low risk' (no thrombosis history, age < 60 years and JAK2-mutated); intermediate risk' (no thrombosis history, age > 60 years and JAK2-wide type) and high risk (thrombosis history or age > 60 years with JAK2 mutation). In 74 patients, 10 (13.5%) were categorized as R-IPSET very low-risk group, 14 (18.9%) low-risk group, 17 (23.0%) intermediate- risk group and 33 (44.6%) high-risk group. JAK2V617F mutations were detected in 53 subjects (71.6%), CALR mutations in 8 (10.8%), MPL mutations in 1 (1.3%), triple-negative (no detectable mutation in JAK2, CALR or MPL) in 9 (12.2%) and other in 3 (4.1%). According to the European consensus, 9 (12.2%) had MF-0, 28 (37.8%) MF-1, 13 (17.6%) MF-2, 17 (23.0%) MF-3 and 7 (9.4%) no data. In this study, splenomegaly mainly occurred in PMF patients, with an incidence rate of 84.0% (21/25) in PMF patients (Table 1).

**Table 1:** Basic information about patients with BCR/ABL-negative MPN

Characteristics	Total (n = 74)	ET (n = 28)	PV (n = 21)	PMF (n = 25)
Sex (male/female)	30/44	(9/19)	(9/12)	(12/13)
Age (year)	65 (32–84)	65 (42–84)	58 (41–73)	68 (32–82)
WBC, median (× 10^9^/L, IQR)	10.2 (0.6–83.6)	6.4 (0.6–19.1)	11.9 (2.5–41.9)	12.1 (0.9–83.6)
> 15 × 10^9^/L, n (%)	21 (28.4)	4 (5.4)	5 (6.8)	12 (16.2)
Hb level, median (g/L, IQR)	102 (50–234)	116 (50–191)	156 (50–234)	87 (50–162)
< 100 g/L, n (%)	35 (47.3)	10 (13.5)	9 (12.2)	16 (21.6)
PLT, median (× 10^9^/L, IQR)	571 (162–958)	863 (462–1051)	374 (214–674)	190 (91–766)
> 300 × 10^9^/L, n (%)	47 (63.5)	23 (82.1)	13 (61.9)	11 (44.0)
*Bone marrow fibrosis grade*				
0	9 (12.2)	8 (28.6)	1 (4.8)	0
1	28 (37.8)	11 (39.3)	10 (47.6)	7 (28.0)
2	13 (17.6)	3 (10.7)	1 (4.8)	9 (36.0)
3	17 (23.0)	4 (14.2)	4 (19.0)	9 (36.0)
No data	7 (9.4)	2 (7.1)	5 (23.8)	0
*Mutation type*				
JAK2V617F	53 (71.6)	20 (71.4)	17 (80.9)	16 (64.0)
CALR	8 (10.8)	3 (10.7)	0	5 (20.0)
MPL	1 (1.3)	0	1 (4.8)	0
Triple negative	9 (12.2)	5 (17.9)	3 (14.3)	1 (4.0)
Other	3 (4.1)	0	0	3 (12.0)
*Thrombotic events*				
Arterial	14 (18.9)	6 (21.4)	4 (19.0)	4 (36.0)
Venous	10 (13.5)	5 (17.9)	1 (4.8)	4 (16.0)
Hemorrhagic events	8 (10.8)	4 (14.2)	1 (4.8)	3 (12.0)
Palpable splenomegaly	27 (36.5)	3 (10.7)	3 (14.3)	21 (84.0)
*R-IPSET category*				
Very low	10 (13.5)	4 (14.2)	4 (19.0)	2 (8.0)
Low risk	14 (18.9)	5 (17.9)	3 (14.3)	6 (24.0)
Intermediate	17 (23.0)	5 (17.9)	3 (14.3)	9 (36.0)
High risk	33 (44.6)	14 (50.0)	11 (52.4)	8 (32.0)

*MPN* confidence interval, *ET* odds ratio, *PV* interquartile range, *PMF* standard deviation, *WBC* white blood cell, *Hb* hemoglobin, *PLT* platelet, *IQR* interquartile range, *R-IPSET* the revised international prognostic score for thrombosis in essential thrombocythemia (IPSET)

### Vascular events in patients with BCR/ABL-negative MPN

A major thrombotic event occurred in 24 patients (32.4%), including 14 arterial (18.9%), and 10 venous (13.5%) thromboses, and the total incidence of thrombotic event in ET, PV and PMF patients was 39.3% (11/28), 23.8% (5/21) and 32.0% (8/25) respectively (Table 1). These patients experienced thrombotic events included cerebral infarction, myocardial infarction, splenic infarction, abdominal thrombosis, coronary artery stenosis, pulmonary embolism, portal thrombosis, and renal vascular thrombosis (Fig. 1). The thrombotic events were mainly cerebral infarction and coronary artery stenosis (the degree of stenosis met the diagnostic criteria for coronary atherosclerotic heart disease), and hemorrhagic events in some patients were associated with thrombotic treatment. There were 8 out of 74 patients (10.8%) experienced bleeding events, with incidence rates of 17.9% (5/28), 4.8% (1/21), and 16.0% (4/25) in ET, PV and PMF patients, respectively (Table 1). Among them, bleeding events were more common in mucosal bleeding, subcutaneous hematoma, gastrointestinal bleeding and cerebral hemorrhage (Fig. 1).

The results of this study showed that patients with thrombotic events had higher fibrinogen (FIB) level (*P* = 0.009), FDP level (*P* = 0.010) and lower ATIII activity (*P* = 0.021), compared to patients without hemorrhagic-thrombotic events. The white blood cell count, hemoglobin level, platelet count, prothrombin time (PT), activated partial thromboplastin time (APTT), D-Dimer, and international standardized ratio (INR) of patients with and without thrombotic events had no differences (*P* > 0.05) (Table 2). Compared with patients who did not experience hemorrhagic-thrombotic events, patients with hemorrhagic events had lower white blood cell count (*P* = 0.030) and hemoglobin level (*P* = 0.003), higher FDP level (*P* = 0.005); and with no differences in platelet count, PT, APTT, FIB, D-Dimer, ATIII, INR (*P* > 0.05) (Table 2). In addition, patients with thrombotic events had higher JAK2V617F gene mutation rate (87.5, 21/24 versus 66.7%, 27/42; *P* = 0.042) compared to patients without hemorrhagic -thrombotic events (Table 2). Among patients with JAK2V617F gene mutation, patients with thrombotic events had higher FIB (*P* = 0.049) and FDP (*P* = 0.015) level, and lower ATIII activity (*P* = 0.006), compare to patients without hemorrhagic- thrombotic events (Table 3). However, compared to JAK2 positive patients with MPN, patients with JAK2 wild type had no statistically significant difference in white blood cell count, hemoglobin level, platelet count, PT, APTT, FIB, D-Dimer, ATIII, INR, etc. (*P* > 0.05) (Table 4).

**Table 2:** Analysis of clinical characteristics and vascular events in patients with BCR/ABL-negative MPN

Characteristics	Thrombotic events (group A, n = 24)	Hemorrhagic events (group B, n = 8)	No hemorrhagic-thrombotic events (group C, n = 42)	A versus C P value	B versus C P value
*Sex, n (%)*				0.346	0.687
Male	8 (33.3)	3 (37.5)	19 (45.2)		
Female	16 (66.7)	5 (62.5)	23 (54.8)		
Age (year), n (%)				0.670	0.779
≥ 60	15 (62.5)	5 (62.5)	24 (57.1)		
< 60	9 (37.5)	3 (37.5)	18 (42.9)		
*Peripheral blood cell count*					
WBC, median (× 10^9^/L, IQR)	10.7 (3.4–83.6)	7.3 (2.8–67.1)	10.2 (0.6–38.8)	0.313	0.030
Hb, median (g/L, IQR)	95 (66–184)	73 (50–86)	120 (50–234)	0.086	0.003
PLT, median (× 10^9^/L, IQR)	674 (3–1308)	119 (2–1811)	613 (24–2239)	0.458	0.076
*Coagulation function*					
PT, median (s, IQR)	12.6 (9.1–19.4)	13.6 (11.8–20.1)	11.9 (9.9–19.2)	0.460	0.070
APTT, median (s, IQR)	30.9 (22.3–48.4)	31.2 (25.5–36.3)	30.5 (23.3–68.9)	0.465	0.345
FIB, median (g/L, IQR)	3.5 (1.1–8.3)	3.3 (2.0–5.0)	2.6 (1.4–6.3)	0.009	0.148
FDP, median (mg/L, IQR)	3.1 (0.4–18.5)	2.2 (0.4–34.6)	1.3 (0.3–7.7)	0.010	0.005
D-Dimer, median (mg/L, IQR)	0.9 (0.1–7.6)	0.8 (0.3–4.2)	0.5 (0.1–12.2)	0.256	0.513
ATIII, median (%, IQR)	75.3 (20.3–118.0)	96.1 (58.9–120.8)	92.5 (47.3–130.0)	0.021	0.801
INR, median (IQR)	1.1 (0.8–1.7)	1.1 (1.0–1.8)	1.1 (0.9–1.7)	0.432	0.169
*A panel of 4 biomarkers*					
TAT, median (ng/ml, IQR)	5.3 (1.2–18.7)	2.5 (1.9–8.4)	2.8 (0.6–11.1)	0.015	0.490
PIC, median (μg/ml, IQR)	1.0 (0.13–2.35)	0.6 ( 0.4–2.0)	0.4 (0.1–1.6)	0.003	0.052
TM, median (TU/ml, IQR)	15.9 (2.8–35.2)	8.7 (2.9–23.6)	10.8 (1.4–27.3)	0.045	0.461
t-PAIC, median (ng/ml, IQR)	9.8 (1.7–26.6)	8.4 (1.3–21.2)	4.9 (1.7–19.6)	0.027	0.054
*JAK2V617F, n (%)*				0.042	0.923
Positive	21 (87.5)	5 (62.5)	27 (66.7)		
Negative	3 (12.5)	3 (37.5)	15 (33.3)		

*MPN* confidence interval, *WBC* white blood cell, *Hb* hemoglobin *PLT* platelet, *IQR* interquartile range, *PT* prothrombin time, *APTT* partial thromboplastin time, *FIB* fibrinogen, *FDP* fibrin/fibrinogen degradation products, *ATIII* Antithrombin III, *INR* international standardized ratio, *TAT* thrombin-antithrombin complex, *PIC* plasmin-α_2_-plasmininhibitor complex, *TM* thrombomodulin, *t-PAIC* tissue plasminogen activator-inhibitor complex

**Table 3:** Comparison of clinical characteristics of patients with JAK2V617F mutated MPN between thrombotic events and no hemorrhagic-thrombotic events

Characteristics	JAK2V617F positive( +)		P value
	Thrombotic events (n = 21)	No hemorrhagic-thrombotic events (n = 27)	
*Age (year), n (%)*			0.173
≥ 60	15 (71.4)	14 (51.9)	
< 60	6 (28.6)	13 (48.1)	
*Peripheral blood cell count*			
WBC, median (× 10^9^/L, IQR)	9.1 (7.3–16.0)	10.1 (6.4–13.0)	0.467
Hb, median (g/L, IQR)	100 (82–133)	128 (82–158)	0.099
PLT, median (× 10^9^/L, IQR)	639 (167–791)	613 (219–964)	0.260
*Coagulation function*			
PT, median (s, IQR)	12.6 (11.6–13.6)	11.8 (10.9–12.2)	0.175
APTT, median (s, IQR)	31.7 (28.4–32.6)	30.8 (27.5–33.1)	0.940
FIB, median (g/L, IQR)	3.5 (2.4–4.1)	1.9 (2.5–3.1)	0.049
FDP, median (mg/L, IQR)	3.8 (1.2–6.3)	1.1 (0.7–2.8)	0.015
D-Dimer, median (mg/L, IQR)	0.9 (0.4–2.1)	0.4 (0.2–0.7)	0.431
ATIII, median (%, IQR)	69.2 (60.8–82.7)	93.5 (82.6–98.9)	0.006
INR, median (IQR)	1.1 (1.0–1.2)	1.0 (0.9–1.1)	0.149
*A panel of 4 biomarkers*			
TAT, median (ng/ml, IQR)	5.5 (2.8–6.8)	2.3 (1.1–3.8)	0.016
PIC, median (μg/ml, IQR)	0.9 (0.4–1.5)	0.4 (0.3–0.7)	0.013
TM, median (TU/ml, IQR)	14.3 (9.7–21.6)	10.3 (7.9–16.6)	0.047
t-PAIC, median (ng/ml, IQR)	9.4 (3.8–15.9)	4.9 (3.4–10.5)	0.079

*MPN* confidence interval, *WBC* white blood cell, *Hb* hemoglobin, *PLT* platelet, *IQR* interquartile range, *PT* prothrombin time, *APTT* partial thromboplastin time, *FIB* fibrinogen, *FDP* fibrin/fibrinogen degradation products, *ATIII* Antithrombin III, *INR* international standardized ratio, *TAT* thrombin-antithrombin complex, *PIC* plasmin-α_2_-plasmininhibitor complex, *TM* thrombomodulin, *t-PAIC* tissue plasminogen activator-inhibitor complex

**Table 4:** Comparison of clinical characteristics of patients with BCR/ABL-negative MPN between JAK2 positive and JAK2 wild type

Characteristics	BCR/ABL-negative MPN		P value
	JAK2 positive (n = 53)	JAK2 wild type* (n = 19)	
*Age (year), n (%)*			0.464
≥ 60	33 (62.3)	10 (52.6)	
< 60	20 (37.7)	9 (47.4)	
*Peripheral blood cell count*			
WBC, median (× 10^9^/L, IQR)	10.3 (5.4–17.1)	8.5 (6.1–12.1)	0.680
Hb, median (g/L, IQR)	112 (82–151)	87 (77–117)	0.341
PLT, median (× 10^9^/L, IQR)	537 (167–879)	540 (119–982)	0.312
*Coagulation function*			
PT, median (s, IQR)	12.0 (11.4–13.1)	12.4 (11.1–12.7)	0.709
APTT, median (s, IQR)	30.9 (27.5–33.0)	30.1 (26.2–32.6)	0.497
FIB, median (g/L, IQR)	3.0 (2.3–4.1)	2.5 (2.0–3.3)	0.883
FDP, median (mg/L, IQR)	2.2 (1.0–4.5)	1.2 (0.6–2.2)	0.789
D-Dimer, median (mg/L, IQR)	0.6 (0.3–1.5)	0.6 (0.3–1.1)	0.824
ATIII, median (%, IQR)	84.6 (68.9–96.9)	92.5 (73.3–102.3)	0.210
INR, median (IQR)	1.1 (1.0–1.2)	1.1 (1.0–1.2)	0.845
*A panel of 4 biomarkers*			
TAT, median (ng/ml, IQR)	3.7 (2.1–6.6)	1.9 (1.2–3.8)	0.013
PIC, median (μg/ml, IQR)	0.6 (0.3–1.0)	0.3 (0.2–0.4)	0.182
TM, median (TU/ml, IQR)	13.8 (9.1–20.6)	4.1 (2.8–7.6)	< 0.001
t-PAIC, median (ng/ml, IQR)	7.3 (3.8–12.8)	3.1 (2.1–6.3)	0.003

*Among the patients without hemorrhagic-thrombotic events, 2 patients′ coagulation function data were missing

*MPN* confidence interval, *WBC* white blood cell, *Hb* hemoglobin, *PLT* platelet, *IQR* interquartile range, *PT* prothrombin time, *APTT* partial thromboplastin time, *FIB* fibrinogen, *FDP* fibrin/fibrinogen degradation products, *ATIII* Antithrombin III, *INR* international standardized ratio, *TAT* thrombin-antithrombin complex, *PIC* plasmin-α_2_-plasmininhibitor complex, *TM* thrombomodulin, *t-PAIC* tissue plasminogen activator-inhibitor complex

### The characteristics of TM, TAT, PIC, and t-PAIC in BCR/ABL-negative MPN patients

At the time of diagnosis, we evaluated TAT, PIC, TM and t-PAIC in the BCR/ABL-negative MPN patients. Among patients with vascular events, significant differences in the four indicators were observed between the patients with thrombotic events and patients without hemorrhagic-thrombotic events, as follows: TAT (median: 5.3 versus 2.8; *P* = 0.015), PIC (median: 1.0 versus 0.4; *P* = 0.003), TM (median: 15.9 versus 10.8; *P* = 0.045), and t-PAIC (median: 9.8 versus 4.9; *P* = 0.027) (Table 2). Compared to the patients without hemorrhagic-thrombotic events, patients with hemorrhagic events had a lower median value in TAT (2.5 versus 2.8) and TM (8.7 versus 10.8), and a higher median value in PIC (0.6 versus 0.4) and t-PAIC (8.4 versus 4.9); however, there is no statistical difference in them (*P* > 0.05) (Table 2). Among patients with JAK2V617F gene mutation, patients with thrombotic events had higher TAT (median: 5.5 versus 2.3; *P* = 0.016), PIC (median: 0.9 versus 0.4; *P* = 0.013) and TM (median: 14.3 versus 10.3; *P* = 0.047) level, and no difference in t-PAIC (median: 9.4 versus 4.9; *P* = 0.079), compare to patients without hemorrhagic-thrombotic events (Table 3). Notablely, JAK2 positive patients with MPN had higher TAT (median: 3.7 versus 1.9; *P* = 0.013), TM (median: 13.8 versus 4.1; *P* < 0.001) and t-PAIC (median: 7.3 versus 3.1; *P* = 0.003) level, and no difference in PIC (median: 0.6 versus 0.3; *P* = 0.182), compared to patients with JAK2 wild type (Table 4).

**Fig. 2:**
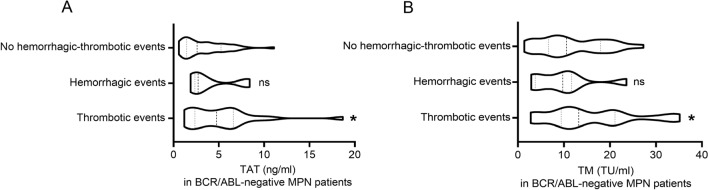
Box plots of TAT values **A** and TM values **B** in BCR/ABL-negative myeloproliferative neoplasms (MPN) patients with thrombotic events, thrombotic events, and without hemorrhagic-thrombotic events. Compared to patients without hemorrhagic-thrombotic events: ^ns^ *P* > 0.05, ^*^P < 0.05; with Mann–Whitney *U* test

**Fig. 3:**
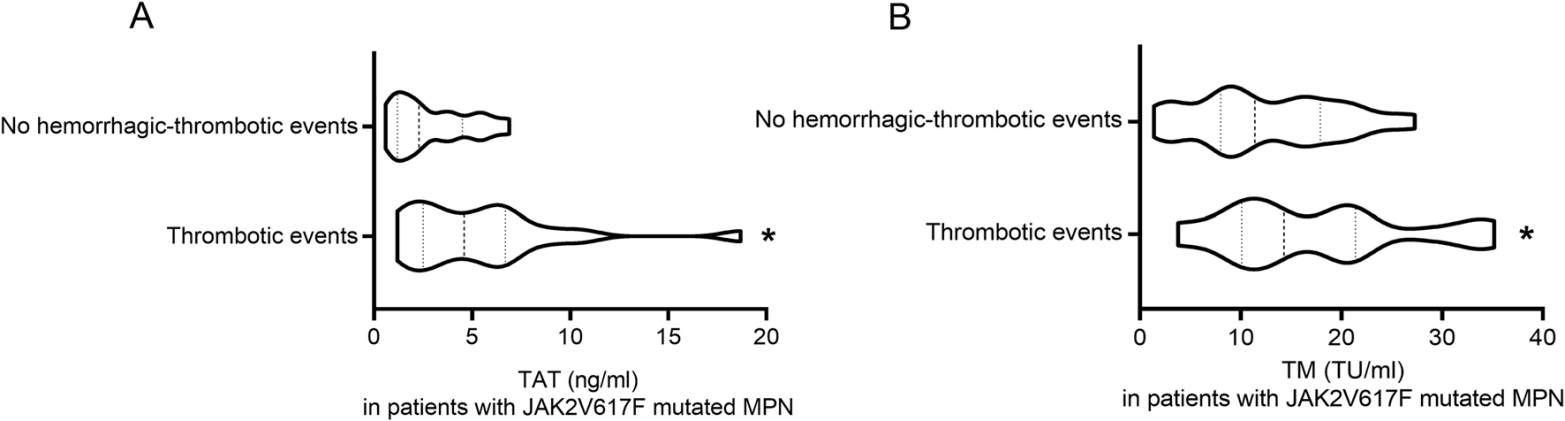
Box plots of TAT values **A** and TM values **B** in JAK2V617F mutated myeloproliferative neoplasms (MPN) patients with thrombotic events and without hemorrhagic-thrombotic events. ^*^P < 0.05; with Mann–Whitney *U* test

**Fig. 4:**
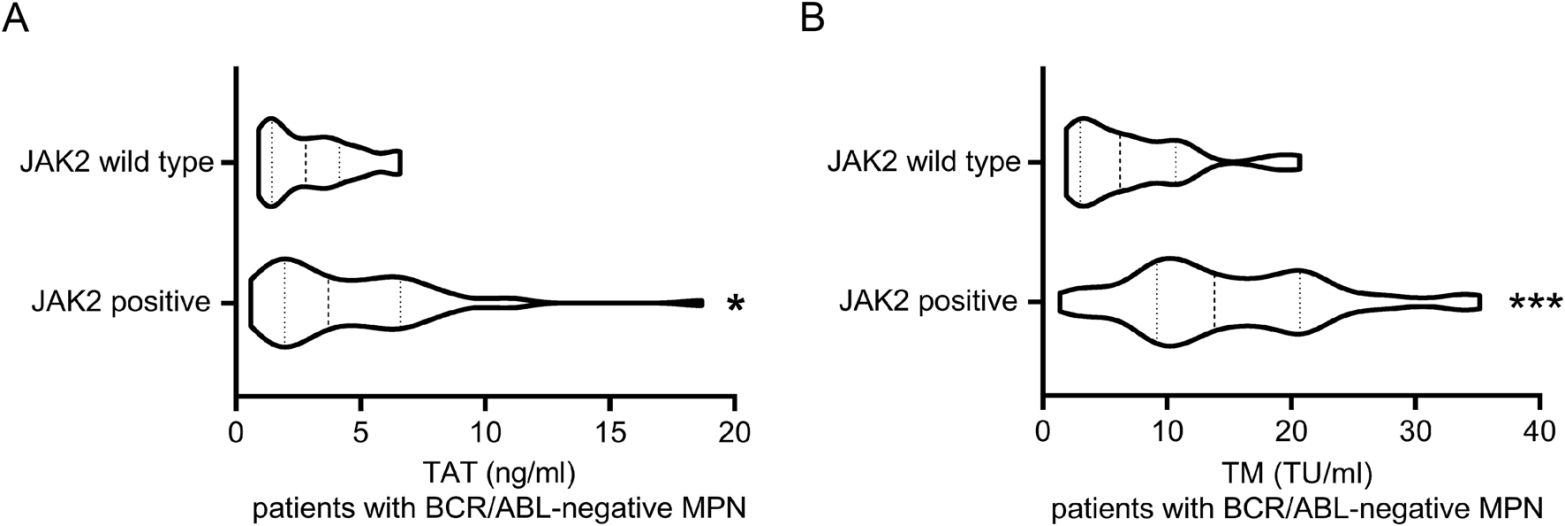
Box plots of TAT values **A** and TM values **B** in BCR/ABL-negative myeloproliferative neoplasms (MPN) patients with JAK2 wide type and JAK2 positive. ^*^P < 0.05, ^***^P < 0.001; with Mann–Whitney *U* test

TAT is recognized as a marker of activation of the coagulation system, and TM can monitor the function of endothelial cells. As they can be significantly elevated before thrombus, so they are important markers of venous thrombosis. Therefore, we analyzed the indicators TAT and TM values for patients (with thrombotic or hemorrhagic events) and controls (patients without hemorrhagic-thrombotic events). In general, TAT and TM values with thrombotic events were higher in patients than controls (*P* < 0.05) (Fig. 2). However, TAT and TM values had no differences in patients with hemorrhagic events than in controls (*P* > 0.05) (Fig. 2). When the analysis was restricted to the JAK2V617F mutated MPN patients, the median TAT and TM values in patients remained significantly higher than in controls (*P* < 0.05) (Fig. 3). As shown in Fig. 4, the median TAT and TM values in patients with BCR/ABL-negative MPN who were JAK2 positive was comparable to that observed in those who were JAK2 wild type (Fig. 4). Furthermore, in the whole study population, the TAT value was positively correlated with thrombotic events (Spearman *r*= 0.287, *P* = 0.019), while the TM value was not (Spearman *r* = 0.223, *P* = 0.073).

## Discussion

Complications such as thrombotic events and hemorrhagic events are the main causes of morbidity and mortality in the BCR/ABL-negative MPN [1, 23]. The occurrence of vascular events seriously affects the prognosis and quality of life of MPN patients [24]. A prospective study showed that the incidence of hemorrhagic and thrombotic events is high in both ET and PV, and these complications have a adverse effect on life expectancy [25]. Thrombosis is a common complication of MPN that significantly impacts patients’ mortality, accounting for the death of 33% of PV, 16% of ET, and 2% of PMF patients [26]. The incidence of thrombotic events in 74 MPN patients included in this study was 32.4%, with a higher incidence in arterial thrombosis than in venous thrombosis, accounting for 18.9 and 13.5%, respectively, which is consistent with the previous studies [27, 28]. Our data has shown that some MPN patients have thrombosis at initial diagnosis. A meta-analysis involving 13,436 MPN patients showed that 9.5–38.6% of initial patients had thrombosis, and the incidence of arterial thrombosis was also higher than venous thrombosis (16.2 versus 6.2%) [29]. Studies have shown that patients with ET and PV have a higher risk of thrombosis before and at diagnosis, while patients with PMF are more likely to have thrombosis after diagnosis [27]. Thrombosis may be the first symptom of MPN, and the initiating treatment is based on thrombosis prediction [30]. Thus, identifying predictive factors for thrombosis is the initial step in MPN patients.

The current research on MPN are related to genes mutations such as JAK2V617F, CALR, and MPL [1]. Cazzola et al. found that JAK2V617F gene mutations are found in 95% of PV patients and 60–65% of ET or PMF patients, CALR gene mutations are found in 20–25% of ET and PMF patients, and MPL gene mutations are found in 5% of ET and PMF patients [31]. The results of this study showed that the incidence of JAK2V617F and CALR gene mutations in MPN patients was similar to the above reports. Age, thrombosis history, cardiovascular factors and JAK2V617F mutation have been identified as predictive factors for thrombosis in MPN patients [30, 32]. It has become apparent that JAK2 V617F confer an independent risk factor for venous thrombosis, but not for arterial thrombosis in ET and PMF [33–35]. The IPSET has been validated to help clinicians estimate the probability of thrombotic events in ET and more recently pre-PMF [7, 36, 37]. However, the current predictive models do not consider hematological responses, hypercoagulable states, or endothelial dysfunction that may be critical for thrombosis [7]. The mechanisms of thrombosis in MPN are complex, and the quantitative and qualitative changes of blood cells play important roles [7, 38]. In fact, patients with ET with extreme thrombocytosis tend to have bleeding rather than thrombotic complications [3, 39], and a recent study found that persistently elevated leukocyte trajectories were not associated with increased thrombosis risk in PV [40]. In addition, neither hematocrit nor platelet count was associated with thrombosis or disease evolution [33, 40]. The activated blood cells and vascular endothelial cells create a highly proadhesive and prothrombotic milieu in the circulation, making MPN patients susceptible to thrombosis [3]. Alterations of plasma thrombotic markers, including increased levels of thrombin-prothrombin complex, contribute to the hypercoagulable state in MPN patients [41]. Although hypercoagulability secondary to blood cell activation has been reported [42], one of the challenges in vascular and thrombosis risk assessment of patients with MPN remains the lack of reliable coagulation biomarkers to supplement the clinical risk factors [7]. Hence, there is a need for more reliable markers to aid clinicians in recommending optimal interventions for MPN patients.

TAT depends on ATIII, and indicates the activation of the coagulation system [43]. Binding of TM with thrombin can reduce the activity of thrombin and enhance the activity of protein C [44]. PIC reflects the balance of the fibrinolysis and anti-fibrinolysis system and the rise of PIC level can indicate the activation of plasmin [45]. Decreased t-PAIC is associated with severe postpartum hemorrhage [19]. Furthermore, Mei et al. found that TAT, PIC, TM, and t-PAIC could serve as effectively diagnostic and prognostic biomarkers of DIC [46]. This study found that higher FIB and FDP level, lower ATIII activity, higher TAT, PIC, TM and t-PAIC, and the JAK2V617F gene mutation are associated with thrombosis in MPN patients. Among them, patients with higher TAT, TM and t-PAIC were more likely to experience thrombotic events, and only TAT was positively correlated with thrombotic events (Spearman *r* = 0.287, *P* = 0.019). Our data showed that the activity of ATIII is significantly lower in MPN patients with thrombosis, which can be explained by pathologically increased thrombin formation and increased consumption for the formation of TAT complexes [47]. In spite of the recognized risk of thrombosis, patients with MPN show little or no abnormalities of traditional coagulation tests such as PT and APTT, perhaps because these are unable to represent the balance between pro- and anti- coagulants nor the effect of platelets and blood cells [32]. In some previous studies, these 4 items were used to predict the possibility of early thrombosis of the patients [20]. Zhou et al. reported that elevated TAT, PIC, TM, t-PAIC, D-dimer, and FDP were sensitive marker in the diagnosis of venous thromboembolism (VTE) patients with malignant tumors [20]. Consistent with our results, TAT, TM and PIC might be sensitive markers of thrombotic status. When thrombosis and bleeding, two contradictory complications, occur in MPN patients at the same time, they will lead to more serious consequences. Therefore, achieving optimal antithrombotic effects while minimizing bleeding events is a major challenge [48].

This study is the first evaluation of the four novel potential predictive markers of thrombosis in MPN disorders. However, there were some limitations in this study. It was a retrospective study and the population of the study was relatively small, lacking a normal healthy control population. Under normal conditions, the level of TAT, PIC, TM, and t-PAIC in our research was determined at diagnosis and lacked post-treatment results. In this study, we have not yet conducted multivariate Cox survival analysis, in future, we will execute a prospective cohort study to explore the applications of these 4 parameters combined with ATIII, and FDP.

## Conclusions

In conclusion, the presence of high TAT, PIC, TM, t-PAIC, ATIII, and FDP in MPN patients were associated with thrombosis. Both markers may be important factors for thrombosis in the settings of BCR/ABL-negative MPN and further prospective studies to confirm these findings are proposed.

## Data Availability

The data of this study are not publicly available because the information can compromise the privacy of research participants. The datasets generated and/or analyzed during the current study are available from the corresponding author on reasonable request.
